# Abnormal Functional Brain Network in Parkinson's Disease and the Effect of Acute Deep Brain Stimulation

**DOI:** 10.3389/fneur.2021.715455

**Published:** 2021-10-14

**Authors:** Zhibao Li, Chong Liu, Qiao Wang, Kun Liang, Chunlei Han, Hui Qiao, Jianguo Zhang, Fangang Meng

**Affiliations:** ^1^Department of Functional Neurosurgery, Beijing Neurosurgical Institute, Capital Medical University, Beijing, China; ^2^Department of Functional Neurosurgery, Beijing Tiantan Hospital, Capital Medical University, Beijing, China; ^3^Beijing Key Laboratory of Neurostimulation, Beijing, China; ^4^Beijing Neurosurgical Institute, Capital Medical University, Beijing, China; ^5^Chinese Institute for Brain Research, Beijing (CIBR), Beijing, China

**Keywords:** brain network, deep brain stimulation, high-density EEG, functional connectivity, graph theory, Parkinson's disease

## Abstract

**Objective:** The objective of this study was to use functional connectivity and graphic indicators to investigate the abnormal brain network topological characteristics caused by Parkinson's disease (PD) and the effect of acute deep brain stimulation (DBS) on those characteristics in patients with PD.

**Methods:** We recorded high-density EEG (256 channels) data from 21 healthy controls (HC) and 20 patients with PD who were in the DBS-OFF state and DBS-ON state during the resting state with eyes closed. A high-density EEG source connectivity method was used to identify functional brain networks. Power spectral density (PSD) analysis was compared between the groups. Functional connectivity was calculated for 68 brain regions in the theta (4–8 Hz), alpha (8–13 Hz), beta1 (13–20 Hz), and beta2 (20–30 Hz) frequency bands. Network estimates were measured at both the global (network topology) and local (inter-regional connection) levels.

**Results:** Compared with HC, PSD was significantly increased in the theta (*p* = 0.003) frequency band and was decreased in the beta1 (*p* = 0.009) and beta2 (*p* = 0.04) frequency bands in patients with PD. However, there were no differences in any frequency bands between patients with PD with DBS-OFF and DBS-ON. The clustering coefficient and local efficiency of patients with PD showed a significant decrease in the alpha, beta1, and beta2 frequency bands (*p* < 0.001). In addition, edgewise statistics showed a significant difference between the HC and patients with PD in all analyzed frequency bands (*p* < 0.005). However, there were no significant differences between the DBS-OFF state and DBS-ON state in the brain network, except for the functional connectivity in the beta2 frequency band (*p* < 0.05).

**Conclusion:** Compared with HC, patients with PD showed the following characteristics: slowed EEG background activity, decreased clustering coefficient and local efficiency of the brain network, as well as both increased and decreased functional connectivity between different brain areas. Acute DBS induces a local response of the brain network in patients with PD, mainly showing decreased functional connectivity in a few brain regions in the beta2 frequency band.

## Introduction

Over the last few decades, the demonstration of alteration in the structural network or functional network *via* neuroimaging data has gained increasing attention in neuroscience and cognitive neuroscience research (1–4). Neuroimaging can non-invasively probe changes in brain function *in vivo*, which helps us to investigate the pathophysiologic deficits caused by neurological disorders. Modern network science including dynamic systems theory, graph theory, statistics, and connectivity analysis has been applied to investigate topological properties of the brain under various states and conditions. Graph theory, a powerful mathematical approach, illustrates a complex network architecture based on the modern theory of networks, which can offer new insights into the structure and function of the brain network, including their architecture, evolution, development, and clinical disorders. In the brain network, the architecture is characterized as a set of nodes (brain regions) connected by edges (3). The nodes and edges defined from neuroimaging data can be used to represent the brain network to study topological properties (organization) and functional connectivity by network-based statistics. Several neuroimaging approaches have been used to demonstrate functional changes of the brain in many conditions such as epilepsy (5), Parkinson's disease (PD) (6), and Alzheimer's disease (7) and have achieved many significant insights.

PD is characterized mainly by the motor symptoms of tremor, rigidity, bradykinesia, and postural instability and is accompanied by various non-motor (non-movement) symptoms such as depression, sleep disturbances, and dementia (8). The main cause of PD is damage to dopaminergic neurons in the substantia nigra, which results in a lack of dopamine in the mesencephalic structures and basal ganglia (8, 9). However, such local nervous tissue deficits often can lead to dysfunction of the global nervous system (10–12). Therefore, neurological dysfunction caused by PD is not only located in the basal ganglia region but also involves the neocortex. For patients with PD experiencing reduced drug efficacy in the middle and late stages of the disease, deep brain stimulation (DBS) therapy is an established treatment method. DBS involves surgical placement of unilateral or bilateral leads (wires) transcranially in the subthalamic nucleus (STN) or the globus pallidus interna (GPi). It can effectively relieve motor and non-motor symptoms, improving the quality of life for patients with PD (13, 14). Previous studies have used several methods including functional magnetic resonance imaging (fMRI), magnetoencephalography (MEG), and standard electroencephalography (EEG) to demonstrate that compared with healthy controls (HC), there are alterations of the topological properties in patients with PD (12, 15–17). Furthermore, DBS can induce local changes in the beta band: cortical–subcortical connectivity changes and attenuation of interhemispheric corticocortical coherence in the sensorimotor areas (12, 18). However, so far PD-related changes in brain connectivity networks have never been investigated using high-density EEG based on connectivity analysis and graph theory. In addition, generally, DBS can immediately improve the symptoms of patients with PD who respond to DBS treatment, but whether there is an acute (within 24 h), large-scale brain network response remains unclear. In the present study, we recorded high-density EEG during an eyes-closed, resting state in HC and patients with PD in the DBS-ON and DBS-OFF states. Furthermore, we adopted source-level EEG to construct the brain network. The objective of this study was to use functional connectivity and graphic indicators to investigate the abnormal brain network topological characteristics caused by PD and investigate the effect of acute DBS on the abnormal brain network topology of patients with PD.

## Materials and Methods

### Participants

We enrolled a total of 41 participants in this study. Twenty-one were age- and gender-matched HC (7 female, age range 52–58 years, mean age 55.9 years; 14 male, age range 51–70 years, mean age 57.8 years). Twenty patients were diagnosed with PD (10 female, age range 51–70 years, mean age 60.2 years; 10 male, age range 50–75 years, mean age 59.6 years). The diagnosis was based on the clinical diagnostic criteria of the U.K. Parkinson's Disease Society Brain Bank. All the patients with PD underwent surgical treatment with DBS targeting the STN. In addition, each patient underwent clinical assessment with the Hoehn and Yahr (H-Y) scale and the Unified Parkinson's Disease Rating Scale III (UPDRS-III) preoperatively (baseline) and the first day after the start of DBS (30 days after surgery). The inclusion criterion for the patients with PD was having a good therapeutic effect with STN-DBS. The exclusion criteria were: (1) typical PD syndrome induced by drugs or metabolic disorders, encephalitis, or other disease represented by similar symptoms (i.e., multiple system atrophy, progressive supranuclear palsy, and Lewy body dementia); (2) history of significant neurological disease or brain surgery; and (3) neuroimaging findings of severe abnormalities or lesions. The study was approved by the Institutional Review Board of Beijing Tiantan Hospital, Capital Medical University, and written informed consent was obtained from all participants.

### EEG Acquisition and Preprocessing

To begin, all the participants were advised to sit in a chair in a comfortable position and relax for 5 min before EEG acquisition. During recording, participants were instructed to keep their eyes closed and remain awake. The resting-state EEG was recorded using a high-density 256-channel system (EGI System 400; Electrical Geodesics, Inc., Eugene, OR). Electrode impedance was kept below 30 kΩ, and 10 min of ongoing EEG data were acquired with a sampling rate of 1,000 Hz. The acquisition reference was Cz. For patients with PD, the EEG acquisition was performed two times. The first time, EEG data were acquired before the DBS was started (DBS-OFF state). The second time, EEG acquisition was performed 24 h after the DBS was started (DBS-ON state). To eliminate the effects of drugs, patients were asked not to take any anti-Parkinsonian drugs during the 12 h prior to the acquisition.

To remove muscle artifacts, the electrodes in the face and neck were removed to reduce to 204 channels. EEG data were split into non-overlapping epochs of 2 s and segments contaminated by artifacts were deleted and bad channels were interpolated. To remove the artifact from DBS, which was 130 Hz, a band-pass filter was used between 1 and 30 Hz. Subsequently, independent component analysis (ICA) was used to remove the ballistocardiographic, myoelectric, and oculomotor artifacts. Thereafter, components related to ballistocardiography, saccadic eye movements, channel noise, and eye blinking were removed based on the waveform, topography, and spectrogram. Finally, for every participant, 5 min of artifact-free EEG data were selected for the next analysis.

### EEG Source Estimation and Source Parcellation

To localize brain sources and reconstruct their time courses, two steps were required. The first step was to construct a head model, which contains information about the electrical and geometrical characteristics of the head. In this step, a template MRI and EEG data were co-registered through identification of the same anatomical landmarks (left and right pre-auricular points and nasion). A realistic head model was built by segmented MRI using FreeSurfer (19) (https://surfer.nmr.mgh.harvard.edu). The lead field matrix was then computed for a cortical mesh with 15,000 vertices using Brainstorm (20) (https://neuroimage.usc.edu/brainstorm/Introduction) and OpenMEEG (http://openmeeg.github.io) (21). The second step was to construct a source model, which provides information about the location and orientation of the dipole sources to be estimated. This step also solved the EEG inverse problem to reconstruct the temporal dynamics of the cortical regions. In this step, we used weighted minimum-norm estimation (wMNE) to reconstruct the dynamics of the cortical sources. Finally, the source-level time series were extracted using the Desikan–Killiany atlas (22), which contains 68 brain regions (Supplementary Table 1).

### Power Spectrum Density Analysis

After obtaining continuous recordings at the source level, the average power over this resting period was estimated in four frequency bands: theta (4–8 Hz), alpha (8–13 Hz), beta1 (13–20 Hz), and beta2 (20–30 Hz). The PSD analysis of the full 300 s rest recording was computed by a standard fast Fourier transform (FFT) approach with the Welch technique and Hanning windowing function (4-s epoch and overlap of 50%). The signal-to-noise ratio (SNR) of each frequency band was estimated by ratio of within band power to out of band power in each group.

### Functional Connectivity Analysis

Functional connectivity was calculated with phase synchronization (PS) between each two brain areas. Although many studies advised to use phase lag index (PLI) to measure functional connectivity because PLI can overcome the influence of volume conduction, PLI is easy to miss linear but functionally meaningful interactions and reduce phase differences under noisy conditions (23). So, in this study, we used the phase-locking value (PLV) to measure PS. PLV is a range between 0 and 1, which shows interactions between two oscillatory time series by quantification of phase relationships. We calculated the PLV at the four frequency bands. Compared with other combinations, the combination of wMNE/PLV is superior (24).

### Network Analysis

In the present study, network analysis was conducted by graph theory, with a series of nodes (brain regions) and edges (connectivity) between nodes. Graph theory is used to extract information from the functional connectivity matrix. Because we used the Desikan–Killiany atlas that contains 68 brain regions to parcellate the brain, we constructed a network with 68 nodes in this study. The result was fully connected, weighted, and undirected networks. The connection strength between every two nodes was defined as their connectivity (range between 0 and 1). The functional connectivity matrices of each subject were constructed over the range of sparsity thresholds between 0.05 and 0.5. Within each group, the minimum network sparsity is when all nodes are connected in the network at the 0.05 threshold value. Network analysis was conducted at two levels: the global level and the edgewise level. For the global level, the following graph metrics were calculated:

#### Characteristic Path Length

The characteristic path length is the average shortest path length between all pairs of nodes in the network and is the most used measure of functional integration. Random and complex networks have short mean path lengths.

#### Global Efficiency (Eglobal)

The global efficiency is the inverse of the average shortest path length and is used to quantify the overall efficiency of information transfer across the whole network (25). It is also used as a measure of functional integration. A higher global efficiency indicates a faster parallel transfer of information in a network and a superior integration of information (26).

#### Clustering Coefficient

The mean clustering coefficient for the network reflects, on average, the prevalence of clustered connectivity around individual nodes, and it is often interpreted as a metric of information segregation in networks. The clustering coefficient quantifies the number of connections that exist between the nearest neighbors of a node as a proportion of the maximum number of possible connections (27).

#### Local Efficiency (Elocal)

The local efficiency is the average efficiency of the local subgraphs, and it measures how efficient communication is among the first neighbors of a given node when it is removed. It is also used as a metric of functional segregation in the network.

For the edgewise level, we used the measure of each of the weights (correlation value) to quantify functional connectivity.

### Data Statistics

Because of the present study's exploratory method with relatively small sample sizes, the group difference in PSD between the HC and patients with PD in DBS-OFF was tested with independent non-parametric permutation tests. The group difference in PSD between patients with PD in DBS-OFF and DBS-ON was tested with paired non-parametric permutation tests. The number of randomizations was 1,000. The group differences of all the graph matrices were tested using an independent *t*-test and paired-test between HC and PD with DBS-OFF and between patients with PD in DBS-OFF and patients with PD in DBS-ON, respectively. The false discovery rate (FDR) with *p* < 0.05 was applied to control for multiple comparisons. The edgewise connectivity was conducted with the network-based statistic (NBS) (28). The independent and paired *t*-test were used to test group differences between HC and patients with PD in DBS-OFF, and between patients with PD in DBS-OFF and DBS-ON, respectively. NBS was used to control the error rate (Edge *p* = 0.05, Component *p* = 0.01, Number of permutations = 5,000). The statistical analyses were performed with GRETNA (29) and NBS. A *p* < 0.05 was considered statistically significant.

## Results

### Demographics and Clinical Variables

There were no significant differences in age and gender between the patients with PD and HC. For patients with PD, the illness duration was 8.2 ± 3.6 years, and there were significant differences in the UPDRS-III scale between the DBS-OFF and DBS-ON states (46.5 ± 9.9 vs. 17.1 ± 9.0, *p* < 0.001). The improvement rate in the UPDRS-III scale rating was 0.63 ± 0.17. These results are listed in Table 1.

**Table 1:** Demographics and clinical variables.

	**Healthy controls**	**PD (DBS-OFF)**	**PD (DBS-ON)**	**Between-group differences**
Gender (F/M)	7/14	10/10	–	*P* = 0.35
Age (years)	57.1 ± 4.1	59.9 ± 6.1	–	*P* = 0.098
Illness duration (years)	–	8.2 ± 3.6	–	–
UPDRS-III	–	46.5 ± 9.9	17.1 ± 9.0	*P* < 0.001
H-Y scale	–	3	–	–

*F, female; M, male; UPDRS, Unified Parkinson's Disease Rating Scale; H-Y, Hoehn and Yahr*.

### Power-Based Topology Analysis

**Figure 1:**
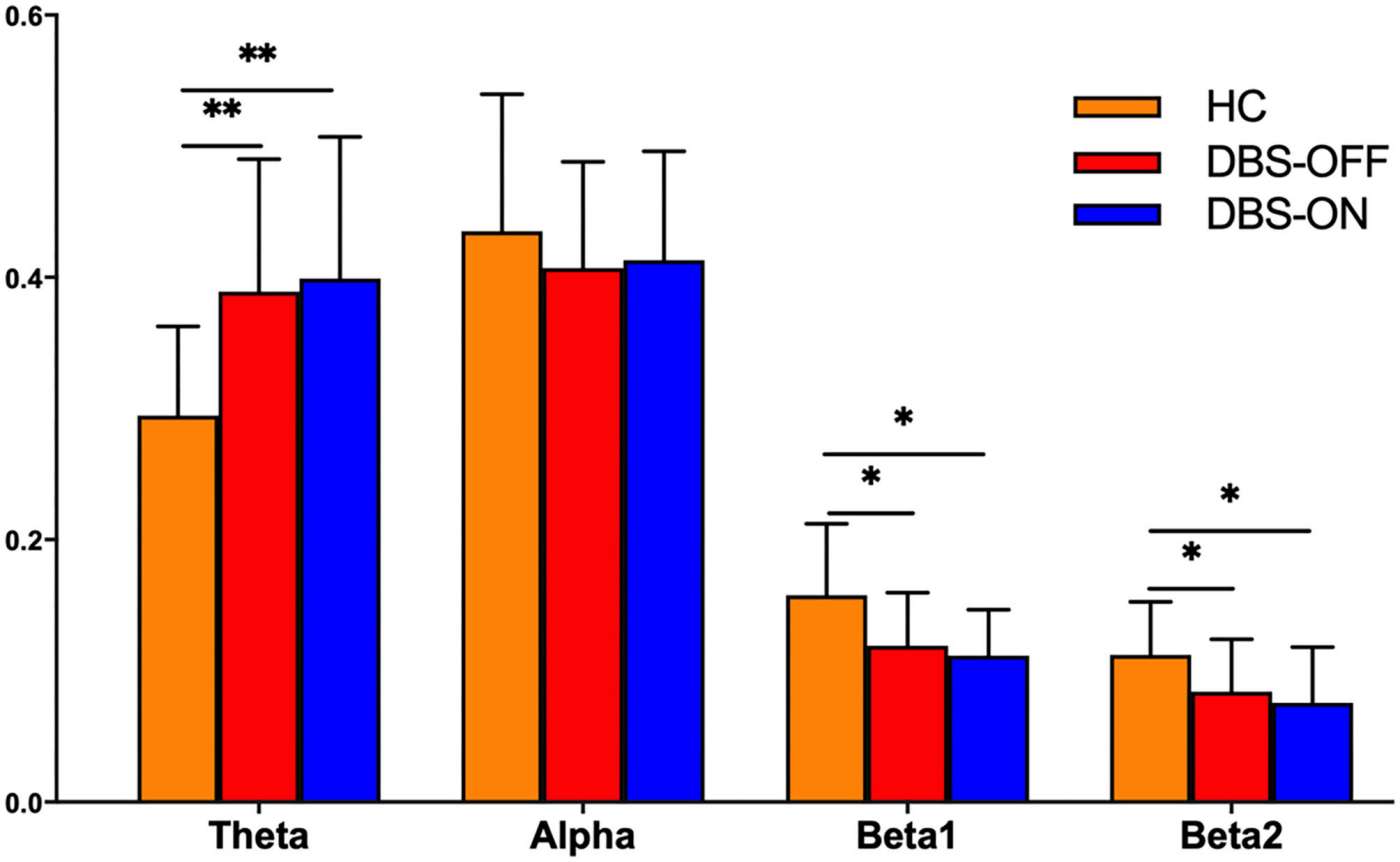
The power-based topology analysis: statistics of power spectral density for each group of patients and healthy controls at four frequency bands: theta (4–8 Hz), alpha (8–13 Hz), beta1 (13–20 Hz), and beta2 (20–30 Hz). The * denotes a *p* < 0.05 and ** denotes a *p* < 0.01.

The results of the frequency-based analysis show that compared with HC, there was a significant increase of PSD in the theta (*p* = 0.003) and decrease of PSD in the beta1 (*p* = 0.009) and beta2 (*p* = 0.04) frequency bands in patients with PD in the DBS-OFF and DBS-ON states. In contrast, there was no difference in the alpha frequency band. However, there were no differences between patients with PD in DBS-OFF and DBS-ON for any frequency bands. In addition, the difference between HC and patients with PT in DBS-ON was similar to HC vs. patients with PD in DBS-OFF. The results are summarized in Figure 1. In addition, the SNR of the theta band of patients with PD was more than HC, and the SNR of the alpha and beta1 bands of HC were more than patients with PD. There was no difference of SNR at the beta2 band in groups (Supplementary Figure 1).

### Global Graph Metrics

**Figure 2:**
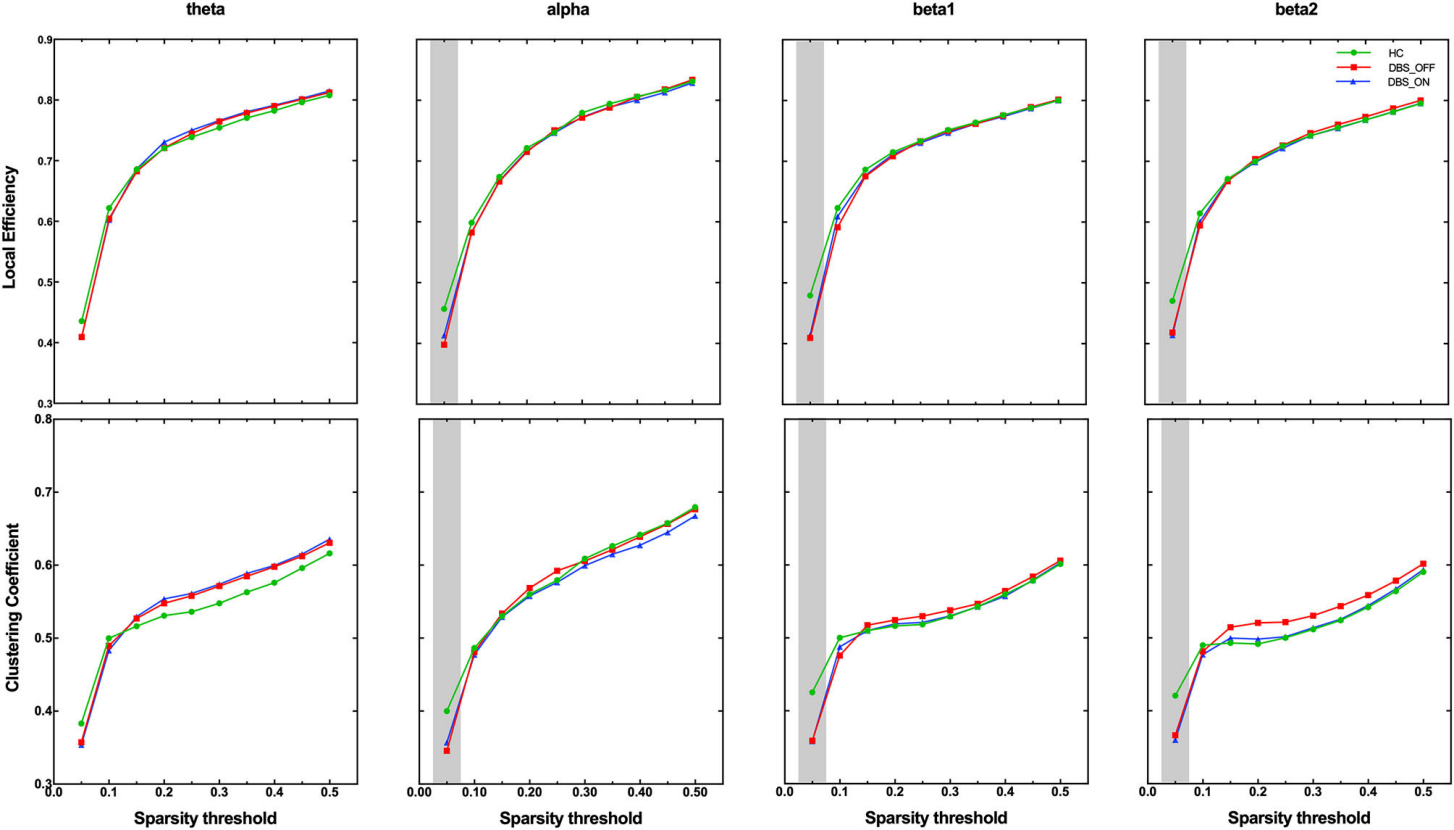
Group comparison of the graphic index across sparsity thresholds. The grey shadings indicate significant group difference at given sparsity thresholds.

On the global level, we analyzed group differences in normalized characteristic path length, global efficiency, clustering coefficient, and local efficiency in all sparsity threshold. Interestingly, among the two parameters that measure the ability of brain network integration, we did not find any difference among groups in characteristic path length and global efficiency. However, we found that the clustering coefficient (*p* < 0.001) and local efficiency (*p* < 0.001), which measure the ability of brain network segregation, of patients with PD in DBS-OFF and DBS-ON decreased significantly for the alpha, beta1, and beta2 frequency bands in lower-density networks (sparsity threshold = 0.05), compared with HC (Figure 2). There were no differences in all global graph metrics between patients with PD in DBS-OFF and DBS-ON in any frequency bands.

### Edgewise Analysis

**Figure 3:**
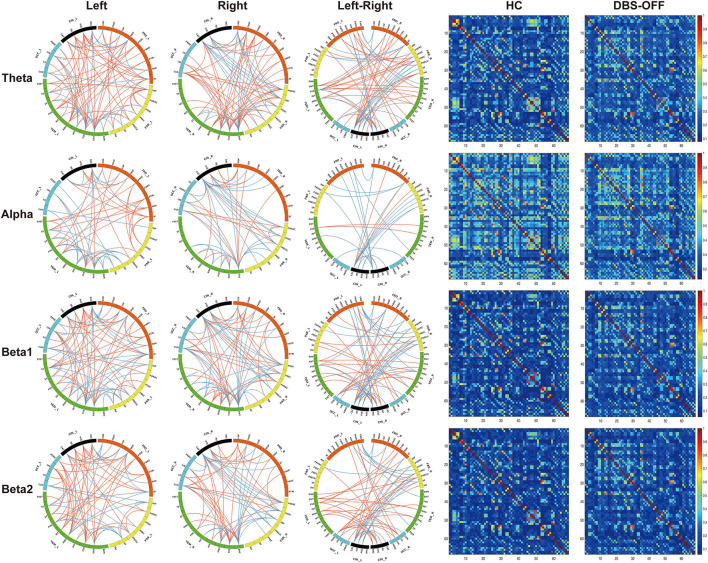
Edgewise significant difference between healthy control and patients with PD in DBS-OFF: the left three columns of circular graphs show significant group differences across groups. The “Left” column shows significant group differences of functional connectivity within the left hemisphere at given frequency bands. The “Right” column shows significant group differences of functional connectivity within the right hemisphere at given frequency bands. The “Left-Right” column shows significant group differences of functional connectivity between the hemispheres at given frequency bands. The orange lines indicate increased functional connectivity, and the blue lines indicate decreased functional connectivity. The right two columns of matrixes indicate the functional connectivity of the group average at given frequency bands.

**Figure 4:**
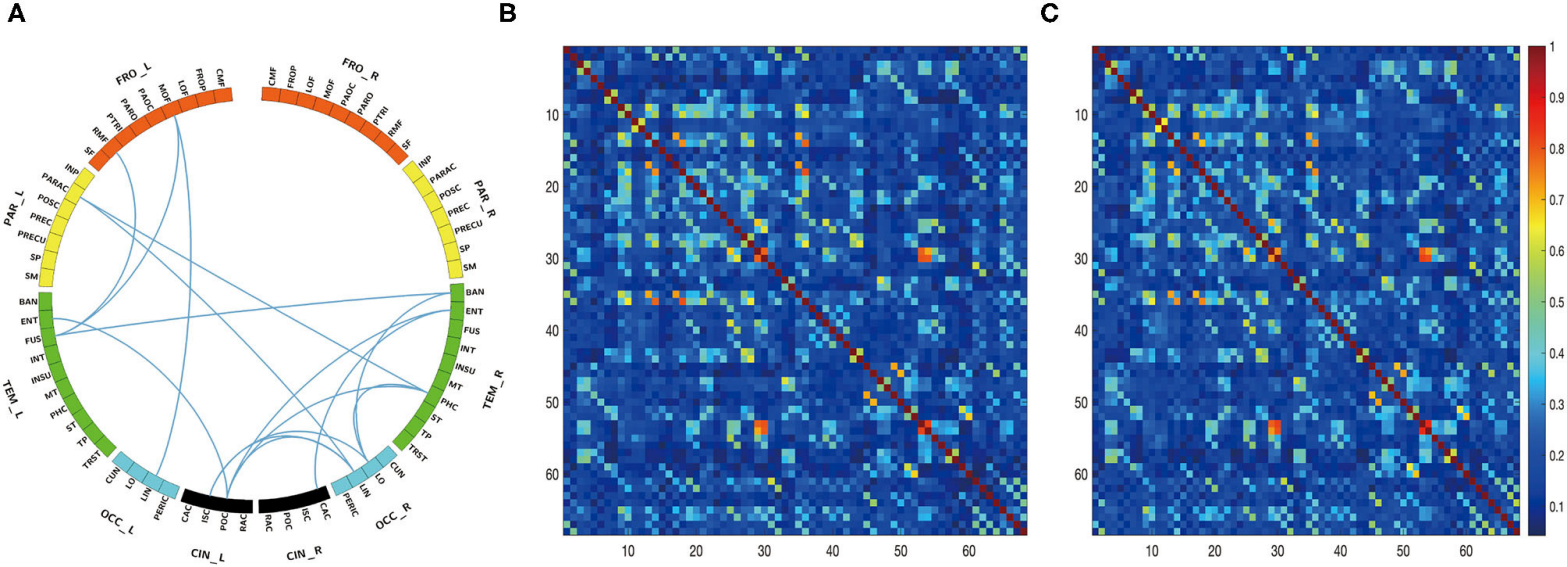
Edgewise significant difference between patients with PD in DBS-OFF and DBS-ON at beta2 frequency band. The circular graph **(A)** shows the group difference of functional connectivity between two groups. The middle matrix **(B)** indicates functional connectivity of the group average of patients with PD in DBS-OFF, and the right matrix **(C)** indicates functional connectivity of the group average of patients with PD in DBS-ON.

The edgewise analysis was conducted using the NBS toolbox, and the results revealed broad-spectrum differences of functional connectivity between HC and patients with PD in DBS-OFF. Significant differences were found at all four analyzed frequencies (Figure 3). However, the difference between DBS-OFF and DBS-ON was slight, only at one frequency band (beta2) (Figure 4).

For the theta network, results only showed significant differences between HC and DBS-OFF. Compared with HC, there were significant decreases of functional connectivity in 85 edges with 50 nodes (*p* < 0.001) and increases of functional connectivity in 207 edges with 68 nodes (*p* < 0.001). In the alpha network, there was a significant difference only between HC and DBS-OFF. Compared with HC, 88 edges with 53 nodes, there was a decrease (*p* < 0.002); however, there was an increase in 91 edges with 51 nodes (*p* = 0.002). For the beta1 network, results showed that compared with HC, 112 edges with 57 nodes, there was a decrease and 156 edges with 64 nodes, there was an increase in patients with PD in DBS-OFF (*p* < 0.001). In the beta2 network, results showed that compared with HC, 84 edges with 45 nodes, there was a decrease (*p* < 0.001) and 168 edges with 61 nodes an increase (*p* < 0.001) in patients with PD in DBS-OFF. In addition, 15 edges with 14 nodes of patients with PD in DBS-ON were lower than in DBS-OFF (*p* = 0.037). To further demonstrate which changes (decrease or increase) were more predominant in functional connectivity analysis between patients with PD and HC, we compared the amounts of increase and decrease in edges as well as the relative node for every frequency band. Although there was no statistically significant difference, the mean of increased connectivity was higher than that for decreased connectivity (92.25 ± 13.28 vs. 155.50 ± 48.20, *p* = 0.074).

## Discussion

In the present study, we used functional connectivity analysis and graph theory based on constructed-source EEG signal to demonstrate that, compared with HC, the patients with PD showed significant decreases in PSD in the theta, beta1, and beta2 frequency bands. In addition, on the global level, clustering coefficient and local efficiency also showed significant decreases in patients with PD for the alpha, beta1, and beta2 frequency bands. On the edgewise level, the functional connectivity of patients with PD was both decreased and increased, but overall, there was a trend toward increased functional connectivity. However, there was a significant difference only in functional connectivity at the beta2 frequency band between patients with PD in the DBS-OFF and DBS-ON states.

### EEG Power and Parkinson's Disease

In early studies, several reports on EEG and MEG studies demonstrated that patients with PD exhibited power changes at multiple frequency bands compared with HC, with theta and alpha1 bands increasing and beta bands decreasing (30–32). Overall, the rhythm of EEG activity in patients with PD is slowing. Moreover, the slowing of resting-state EEG background activity is positively associated with disease progression (33) and negatively associated with cognition (34, 35). In longitudinal EEG studies, lower peak frequency and higher delta/theta power were the best predictors for future conversion to PD dementia (36, 37). In this study, although we did not find alpha frequency band increase, the significant increase of theta frequency and decrease of beta frequency bands were in line with previous studies. The slowing activity of EEG is interpreted as decreased flexibility in brain activity.

### Brain Network of Parkinson's Disease

Network-wide changes in PD are consistently reported, and earlier studies demonstrated that functional connectivity increased with disease progression in the 4–30 Hz range (30, 38). In addition, Bosboom et al. (39) showed that the functional connectivity of different brain areas increased in multiple frequency bands including theta (4–8 Hz), alpha (8–13 Hz), and beta (13–30 Hz) bands in patients with PD without dementia compared with healthy people. In the present study, we found that although the increased functional connectivity seems to dominate, there were also many areas of decreased functional connectivity. This result was consistent with results of a previous study in which the functional connectivity of patients with PD initially increased but decreased over time in relation to disease progression—especially for cognitive decline (40). This finding suggests that the functional connectivity changes in patients with PD are complex and dynamic with disease progression.

Graph theory can be used to evaluate macroscopic brain connections on the local or global levels. A previous study showed that compared with HC, patients with early-stage PD showed decreases in local clustering with a preserved path length in the delta frequency band (17). In addition, longitudinal analysis over 4 years in patients with PD revealed that local clustering progressively decreased in multiple frequency bands together with a decrease in path length in the alpha 2 range. Moreover, the longitudinal brain network changes were associated with attenuation of cognitive and motor function (17). In the present study, although we did not find that there were significant changes in functional integration (global efficiency and characteristic path length) of the brain network, we found a significant decrease in clustering coefficient and local efficiency, which indicated decreased brain network segregation in patients with PD, and the brain networks of patients with PD move toward a more random network organization compared with healthy people. Moreover, our results were not completely consistent with previously published studies; this may be because of a difference in patients' clinical manifestations, disease progression, or measurement methods of the brain network. Further, the topological characteristics in the brain network may show dynamic changes over the whole course of PD, and so the study of brain networks of patients with PD at a single time point cannot provide enough information on topological characteristics.

### The Effect of DBS on Brain Network in PD

Although the effect of DBS on the brain network has been studied, the number of studies is still small. Most studies have focused on the basal-ganglia-cortical motor circuits and cerebello-thalamo-cortical circuits (41). A movement-related potential study had shown interhemispheric cortico-cortical coherence in the beta band was significantly reduced between the bilateral sensorimotor areas in the DBS-ON state (18). Horn et al. (16) demonstrated that effective DBS can increase overall connectivity in the motor network, normalize the network profile toward HC, and specifically strengthen thalamo-cortical connectivity while reducing striatal control over basal ganglia and cerebellar structures. In the present study, although the motor symptoms of patients with PD were significantly relieved, the brain network changes caused by acute DBS were slight; the functional connectivity only decreased in the beta2 frequency band. This finding can be explained by the fact that the EEG recording of patients with PD in DBS-ON was made 24 h after DBS began taking effect, which might not be enough time for the brain to produce significant network changes. In contrast to previous studies (18, 42), this study focused on the therapeutic effect of acute DBS. The formation of the brain network is the result of persistent effects. Okun (43) noted that DBS acted on the cells and fibers around the electrode to inhibit cells and excite fibers (44, 45); furthermore, there were changes in the firing rate and pattern of individual neurons in the basal ganglia (46). Meanwhile, DBS also acts at synapses and triggers neighboring astrocytes to promote the release of calcium and neurotransmitters as well as increase local cerebral blood flow (47–50). Finally, DBS induces local and possibly distal proliferation of neural precursor cells. The long-term effects of these actions will eventually lead to large-scale network changes. Therefore, acute DBS may only induce local brain network changes, which will gradually expand over time until the whole brain network is affected.

### Methodological Considerations

Brain network analysis is a helpful tool to explore both normal and abnormal brain activity. The development of imaging techniques such as MEG, fMRI, and EEG has greatly promoted studies of the brain network. Scalp-level EEG signals are not recommended for use in brain network analysis (51); in contrast, the use of source-level EEG signals to analyze the brain network is advised (52). There are two main issues with scalp-level EEG analysis of functional connectivity: (1) the location of EEG channels cannot accurately reflect brain activity at the source level, and (2) because of the existence of effects of field spread and volume conduction (52), spurious estimates of functional connectivity may occur between channels, where more than one channel can pick up the activity of an underlying source or one channel can pick up more than one underlying source. However, the network representation based on source-level EEG is a better approximation of the unknown true network organization, and the source estimate itself has the effect of reducing volume conduction. Another study suggested using phase lag index (PLI) to calculate functional connectivity to remove volume conduction; however, its risk of missing linear but functionally meaningful interactions and reducing phase differences under noisy conditions may result in the attenuation of the existing difference between HC and patients with PD or the existing difference between patients with PD in DBS-OFF and DNS-ON. This is unfavorable for estimating the difference in local functional connectivity between patients with PD in DBS-ON and DBS-OFF. In this study, the choice of wMNE/PLV was supported by two comparative analyses (24, 53) that demonstrated the superiority of wMNE/PLV over other combinations of five inverse algorithms and five connectivity measures. This method was first used to reveal relevant networks in a picture-naming task (53) and was then extended to the functional connectivity disruption of PD dementia (54) and the tracking of the spatiotemporal dynamics of reconstructed brain networks (55).

### Study Limitations

Although there were some significant changes in patients with PD in the DBS-OFF and DBS-ON states, several limitations existed in this study. First, as the EEG recordings of patients with PD were made 1 month after surgery, the readings might have been affected by a postoperative stun effect, which was likely to associate with a reduction in spontaneous beta activity in the STN and temporary amelioration of Parkinsonism (56). Although the local edema in STN might fade away, local neuron lesions are permanent. Second, the sample size was relatively small; a larger cohort may yield more significant and robust results. Third, we did not consider the relationship between brain network changes and cognitive level. Dementia is one of the most common and important non-motor symptoms encountered in advanced PD (57). Previous studies demonstrated that the PSD and brain network changes are associated with the cognitive level (54, 58–60). Fourth, although we took a set of strict measures to reduce an artifact of EEG data, there were still differences of SNR between groups. On one hand, it is because of the inherent shortcoming of EEG. Daniel and his colleagues have demonstrated that, compared with MEG, the SNR of deep sources was large, however, the SNR of superficial sources was lower (61). On the other hand, pathological movement of patients with PD may influence the SNR of EEG data. Finally, our study only involved STN-DBS instead of GPi-DBS. Like the STN, the GPi is a common therapeutic target for PD. A recent study demonstrated that STN-DBS modulates two distinct neurocircuits, named the GPi-thalamus-deep cerebellar nuclei circuit and the M1-putamen-cerebellum circuit (62). This suggests STN-DBS and GPi-DBS have different effects on brain networks.

In conclusion, compared with HC, patients with PD showed characteristic slowing of EEG background activity, decreased clustering coefficient, and local efficiency of the brain network as well as both increased and decreased functional connectivity between different brain areas. Acute DBS only induces a local response in the brain network of patients with PD, mainly showing decreased functional connectivity in a few brain regions of the beta2 frequency band.

## Data Availability Statement

The raw data supporting the conclusions of this article will be made available by the authors, without undue reservation.

## Ethics Statement

The studies involving human participants were reviewed and approved by the Institutional Review Board of Beijing Tiantan Hospital, Capital Medical University. The patients/participants provided their written informed consent to participate in this study.

## Author Contributions

FM, HQ, and ZL: conception and design. ZL, QW, CL, and KL: acquisition of data. CH and ZL: analysis and interpretation of data. ZL: drafting the article, reviewed submitted version of manuscript, and statistical analysis. CH: critically revising the article. JZ: study supervision. All authors contributed to the article and approved the submitted version.

## Funding

The main support funds were from the National Natural Science Foundation of China (81971070) and Beijing Municipal Administration of Hospitals Clinical medicine Development of special funding support (XMLX201833).

## Conflict of Interest

The authors declare that the research was conducted in the absence of any commercial or financial relationships that could be construed as a potential conflict of interest.

## Publisher's Note

All claims expressed in this article are solely those of the authors and do not necessarily represent those of their affiliated organizations, or those of the publisher, the editors and the reviewers. Any product that may be evaluated in this article, or claim that may be made by its manufacturer, is not guaranteed or endorsed by the publisher.
