# Supplementary material for: Abnormal Functional Brain Network in Parkinson's Disease and the Effect of Acute Deep Brain Stimulation
Source: Front Neurol. 2021 Oct 14;12:715455. doi: 10.3389/fneur.2021.715455 (PMC8551554; doi:10.3389/fneur.2021.715455)
Supplement: Supplementary file 1 [file Data_Sheet_1.PDF]

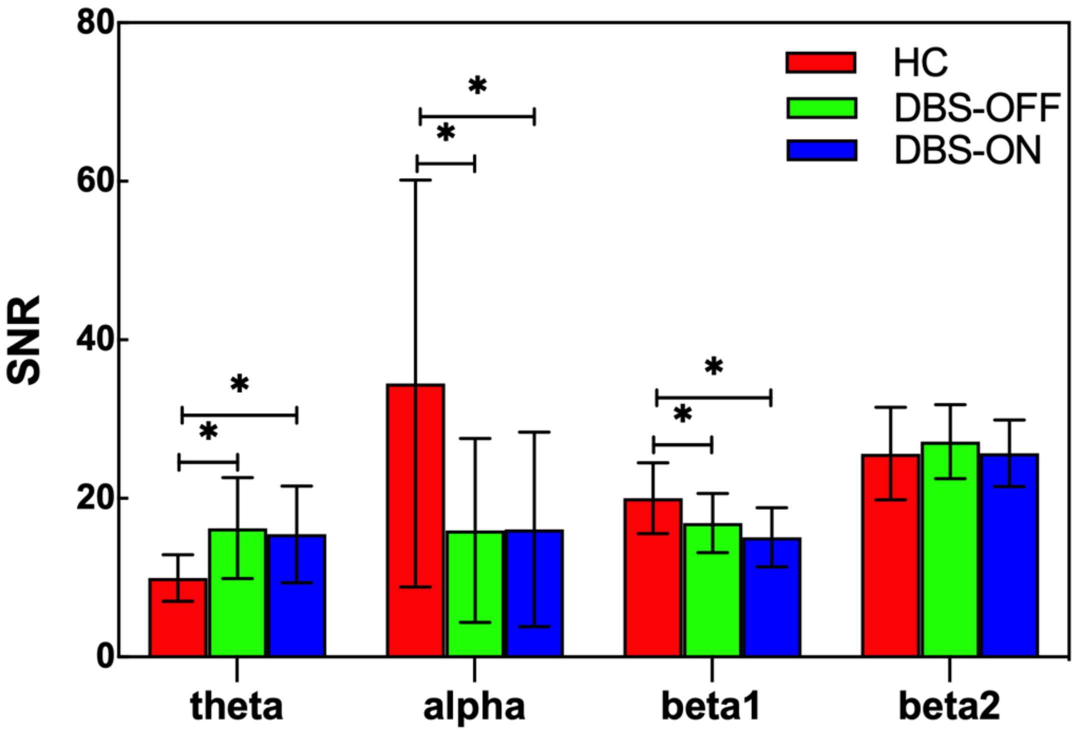

Supplementary Figure 1. The SNR of each frequency band in each group. The SNR of theta band of patients with PD was more than HC, and the SNR of alpha and beta1 band of HC were more than patients with PD. There was no difference of SNR at beta2 band in groups.

Supplementary Table 1. The 68 brain regions based on Desikan-Killiany atlas.

Number	Lable	Abbreviation	Broad regions	Number	Lable	Abbreviation	Broad regions
**1**	bankssts_L	BAN	Tem	2	bankssts_R	BAN	Tem
**3**	caudalanteriorcingulate_L	CAC	Cin	4	caudalanteriorcingulate_R	CAC	Cin
**5**	caudalmiddlefrontal_L	CMF	Fro	6	caudalmiddlefrontal_R	CMF	Fro
**7**	cuneus_L	CUN	Occ	8	cuneus_R	CUN	Occ
**9**	entorhinal_L	ENT	Tem	10	entorhinal_R	ENT	Tem
**11**	frontalpole_L	FROP	Fro	12	frontalpole_R	FROP	Fro
**13**	fusiform_L	FUS	Tem	14	fusiform_R	FUS	Tem
**15**	inferiorparietal_L	INP	Par	16	inferiorparietal_R	INP	Par
**17**	inferiortemporal_L	INT	Tem	18	inferiortemporal_R	INT	Tem
**19**	insula_L	INSU	Tem	20	insula_R	INSU	Tem
**21**	isthmuscingulate_L	ISC	Cin	22	isthmuscingulate_R	ISC	Cin
**23**	lateraloccipital_L	LO	Occ	24	lateraloccipital_R	LO	Occ
**25**	lateralorbitofrontal_L	LOF	Fro	26	lateralorbitofrontal_R	LOF	Fro
**27**	lingual_L	LIN	Occ	28	lingual_R	LIN	Occ
**29**	medialorbitofrontal_L	MOF	Fro	30	medialorbitofrontal_R	MOF	Fro
**31**	middletemporal_L	MT	Tem	32	middletemporal_R	MT	Tem
**33**	paracentral_L	PARAC	Par	34	paracentral_R	PARAC	Par
**35**	parahippocampal_L	PHC	Tem	36	parahippocampal_R	PHC	Tem
**37**	parsopercularis_L	PAOC	Fro	38	parsopercularis_R	PAOC	Fro
**39**	parsorbitalis_L	PARO	Fro	40	parsorbitalis_R	PARO	Fro
**41**	parstriangularis_L	PTRI	Fro	42	parstriangularis_R	PTRI	Fro
**43**	pericalcarine_L	PERIC	Occ	44	pericalcarine_R	PERIC	Occ
**45**	postcentral_L	POSC	Par	46	postcentral_R	POSC	Par
**47**	posteriorcingulate_L	POC	Cin	48	posteriorcingulate_R	POC	Cin
**49**	precentral_L	PREC	Par	50	precentral_R	PREC	Par
**51**	precuneus_L	PRECU	Par	52	precuneus_R	PRECU	Par
**53**	rostralanteriorcingulate_L	RAC	Cin	54	rostralanteriorcingulate_R	RAC	Cin
**55**	rostralmiddlefrontal_L	RMF	Fro	56	rostralmiddlefrontal_R	RMF	Fro
**57**	superiorfrontal_L	SF	Fro	58	superiorfrontal_R	SF	Fro
**59**	superiorparietal_L	SP	Par	60	superiorparietal_R	SP	Par
**61**	superiortemporal_L	ST	Tem	62	superiortemporal_R	ST	Tem
**63**	supramarginal_L	SM	Par	64	supramarginal_R	SM	Par
**65**	temporalpole_L	TP	Tem	66	temporalpole_R	TP	Tem
**67**	transversetemporal_L	TRST	Tem	68	transversetemporal_R	TRST	Tem

Fro = frontal; Tem = temporal; Para = parietal; Occ = occipital; Cin = cingulate.
